# Thromboelastometry versus free-oscillation rheometry and enoxaparin versus tinzaparin: an in-vitro study comparing two viscoelastic haemostatic tests’ dose-responses to two low molecular weight heparins at the time of withdrawing epidural catheters from ten patients after major surgery

**DOI:** 10.1186/s12871-015-0145-2

**Published:** 2015-11-24

**Authors:** Owain Thomas, Anna Larsson, Nahreen Tynngård, Ulf Schött

**Affiliations:** 1 Medical Faculty, University of Lund, Lund, Sweden; 2 Department of Paediatric Anaesthesia and Intensive Care, SUS Lund University Hospital, Lund, Sweden; 3 Department of Clinical Immunology and Transfusion Medicine, Department of Clinical and Experimental Medicine, Linköping University, Linköping, Sweden; 4 Department of Clinical Chemistry, Department of Clinical Experimental Medicine, Linköping University, Linköping, Sweden; 5 Department of Anaesthesia and Intensive Care, SUS Lund University Hospital, Lund, Sweden

**Keywords:** Coagulation, Factor Xa, Thromboelastometry, Free-oscillation rheometry, Low molecular weight heparin, Postoperative, Enoxaparin, Tinzaparin, Epidural haematoma, Spinal haematoma

## Abstract

**Background:**

Monitoring low molecular weight heparins (LMWH’s) in the perioperative period is prudent in patients at high risk of coagulative complications, especially when the patient has an epidural catheter requiring withdrawal, which is associated with the risk of spinal haematoma. The aim of this study was to evaluate the *in vitro* dose-responses of two different LMWH’s on two different viscoelastic haemostatic tests, using blood sampled from patients with normal routine coagulation parameters, on the day after major surgery when their epidural catheters were due to be withdrawn.

**Methods:**

Enoxaparin or tinzaparin were added *in vitro* to blood from ten patients who had undergone oesophageal resection, to obtain plasma concentrations of approximately 0, 0.5, 1.0 and 1.5 IU/mL. Coagulation was monitored using thromboelastometry (ROTEM®) using the InTEM® activating reagent; and free oscillation rheometry (FOR: ReoRox®), activated using thromboplastin. Clot initiation was measured using ROTEM-CT, ReoRox-COT1 and ReoRox–COT2. Clot propagation was measured using ROTEM-CFT, ROTEM-Alpha Angle and ReoRox-Slope. Clot stability was measured using ROTEM-MCF and ReoRox-G’max, and clot lysis was measured using ROTEM-ML and ReoRox-ClotSR.

**Results:**

Clot initiation time assessed by thromboelastometry and FOR was prolonged by increasing concentrations of both LMWH’s (*P* < 0.01). Equivalent doses of tinzaparin in international units (anti-FXa units) per millilitre prolonged clot initiation more than enoxaparin (*P* < 0.05). There was significant inter-individual variation – the ranges of CT and COT1 at LMWH-concentrations of 0 and 1.5 IU/mL overlapped. None of the tests reflecting clot formation rate or stability showed a dose–response to either LMWH but clot lysis showed a tentative negative dose–response to the LMWH’s.

**Conclusions:**

Clot initiation time’s dose-dependent prolongation by LMWH’s in this study agrees with previous research, as does tinzaparin’s stronger anti-coagulative effect than enoxaparin at equivalent levels of anti-FXa activity. This casts doubt on the validity of using anti-FXa assays alone to guide dosage of LMWH’s. The significant inter-individual variation in dose–response suggests that the relationship between dose and effect in the postoperative period is complicated. While both ROTEM and FOR may have some role in postoperative monitoring, more research is needed before any conclusion can be made about their clinical usefulness.

**Electronic supplementary material:**

The online version of this article (doi:10.1186/s12871-015-0145-2) contains supplementary material, which is available to authorized users.

## Background

When low molecular weight heparins (LMWH’s) were first used in clinical practice, monitoring was considered unnecessary [1], but this has recently been questioned since major haemorrhagic complications are regularly reported in patients treated with LMWH and the optimal LMWH dose in the aged, patients with obesity and renal insufficiency is not well defined [2–4]. Hypercoagulative states are common in many settings: postoperative, critical illness, obstetrics, oncology and coronary care; such that ordinary doses of LMWH are insufficient, but overdosing of thrombosis prophylaxis is also dangerous since it predisposes to haemorrhagic complications such as spinal haemorrhage in conjunction with withdrawing an epidural catheter [4–8].

Low molecular weight heparins have more predictable pharmacokinetic and pharmacodynamic properties than unfractionated heparin (UFH), and have therefore become the gold standard in many clinical situations such as thromboprophylaxis, and treatment of deep vein thrombosis (DVT) and pulmonary embolism (PE). Variation in the anticoagulant potency of the numerous LMWH’s that are available is the result of different degrees of inhibition of coagulation factors Xa and IIa. LMWH’s with greater molecular weight are more similar to unfractionated heparin [1, 9, 10]. UFH (mean molecular weight, MW, 15 kDa) inhibits factor Xa and IIa equally whereas enoxaparin (mean MW 4.2 kDa) is a LMWH with a high anti-FXa/anti-FIIa ratio: it inhibits factor Xa four times as strongly as IIa. Tinzaparin (mean MW 6.8 kDa) is more similar to unfractionated heparin in that it inhibits factor Xa only twice as strongly as factor IIa [11].

Routine laboratory plasma based coagulation tests for monitoring heparinization, such as the activated partial thromboplastin time (aPTT), and the chromogenic anti-FXa test only detect changes in the initiation phase of coagulation and are not always rapidly available at all times of the day. It is possible to run viscoelastic haemostatic tests (VHT’s) in ‘patient-near’ laboratories or even bedside at any time of the day, providing preliminary results within minutes and complete results within an hour of blood sampling. There are several commercially available VHT’s which allow analysis not only of the propagation and amplification phases of whole blood coagulation, but also of fibrinolysis and clot structure, which depend upon fibrin polymerization and platelet activity [12, 13].

It would be advantageous to be able to titrate LMWH doses using viscoelastic tests to reduce complications caused by bleeding and thrombosis. However, there are few studies in this area and to our knowledge there are no studies concurrently comparing different LMWH’s with different anti-FXa/anti-FIIa ratios, using different VHT’s [14–16].

The aim of this study was to evaluate dose–response effects of enoxaparin and tinzaparin on ROTEM® and FOR: can these instruments be used to monitor LMWH’s at and above levels used for thrombosis prophylaxis? Our hypothesis was that FOR would be more sensitive to LMWH’s effects on clot formation and strength than thromboelastometry.

## Methods

### Study subjects and sampling

Ten patients who had undergone oesophageal resection were included in the study after informed and signed consent. The study was approved by the local ethics committee in Lund (DNR 2010/482-100).

Blood was sampled from each patient’s indwelling central venous catheter on the day that their epidural catheter was removed, using 4.5 mL BD Vacutainer® citrate tubes. All patients had been routinely sampled the day before to assure normal renal function (creatinine, urea), routine coagulation parameters: activated partial thromboplastin time (aPTT), prothrombin time international normalized ratio (PT-INR) and platelet count (PLT). All patients had received standard thrombosis prophylaxis with enoxaparin 40 mg at 8 p.m. the evening before blood sampling, which took place between 10 a.m. and 2p.m, 14–18 h after the last dose of enoxaparin.

### Titration of blood with LMWH

Enoxaparin (Klexane, Sanofi-Aventis, Guildford, UK) and tinzaparin (Innohep, Leo Pharma, Ballerup, Denmark) were diluted with isotonic saline (9 mg/mL NaCl: Fresenius Kabi, Bad Homburg, Germany) to concentrations of 10, 20 and 30 IU/mL. 60 μL aliquots of saline containing 0, 10, 20 or 30 IU/mL enoxaparin or tinzaparin were then added to 2 mL portions of pre-warmed (37 ° C) citrated blood from each patient to obtain plasma concentrations of 0, 0.5, 1.0 and 1.5 IU/mL of enoxaparin and tinzaparin, respectively, assuming that the blood samples had a haematocrit of 40 %. The samples were incubated for 10 min at 37 °C.

The concentrations of LMWH between 0 and 1.5 IU/ml in this study encompass both thromboprophylactic levels of 0.2-0.4 IU/mL, and higher anti-FXa levels that are above recommended levels [17].

### Viscoelastic coagulation analysis

Clot formation and lysis was studied using thromboelastometry (ROTEM®, Pentapharm, Munich, Germany) and FOR (ReoRox G_2_®_,_ MediRox, Nyköping, Sweden). Analyses were run at 37 °C within 1 h of sampling.

#### Thromboelastometry

**Fig. 1:**
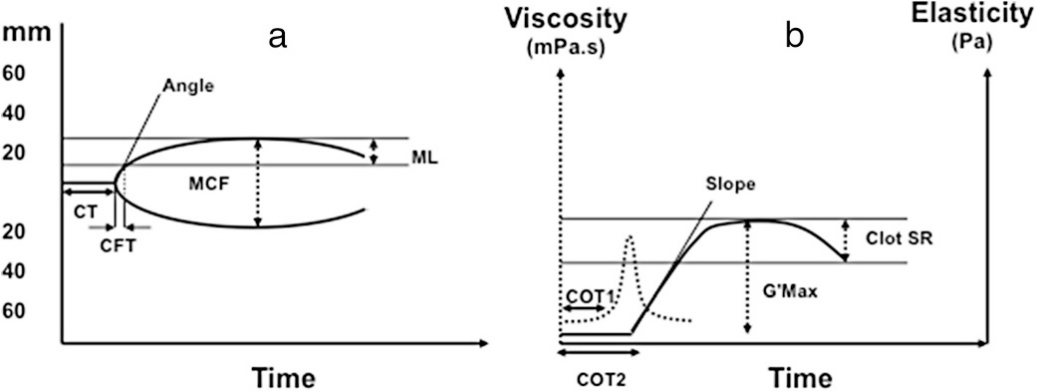
Diagram showing the parameters recorded from rotational thromboelastometry (ROTEM) and free oscillation rheometry (FOR, ReoRox). (**a**) ROTEM. (**b**) ReoRox. A brief explanation of the parameters follows: measures of clot initiation: ROTEM-CT (clot time) and FOR-COT1 and -COT2. Measures of clot propagation: ROTEM-CFT and –alpha angle; and FOR-(COT2-COT1) and –Slope. Measures of clot structure: ROTEM-MCF and FOR-G’max. Measures of fibrinolysis: ROTEM-ML and FOR-Clot SR

Technical details on ROTEM have been described previously [18, 19]. Briefly, the ROTEM® has a fixed sample cup with a pin suspended in the blood sample. The pin oscillates and the movement is registered in the coagulating sample [18]. Analysis of coagulation with ROTEM gives rise to a curve from which the clotting time (CT), clot formation time (CFT), alpha angle, maximum clot firmness (MCF) and maximum clot lysis (ML), which represents fibrinolysis, can be determined as shown in Fig. 1 [19].

After addition of 20 μL of 0.2 M CaCl_2_ (the ‘star-tem®’ reagent) to 300 μL of each sample, coagulation was initiated in each sample by addition of 20 μL of the InTEM® reagent, which contains partial thromboplastin phospholipid and ellagic acid.

#### Free oscillation rheometry (FOR)

FOR was assessed with the ReoRox G_2_ rheometer (MediRox AB, Nyköping, Sweden). The sample is added to a reaction chamber which consists of a gold-coated sample cup with a gold-coated cylinder suspended in the blood sample [20]. The sample cup oscillates and the changes in the frequency and damping of the oscillation in the coagulating sample are registered. Changes in damping give rise to a viscosity curve measured in Pascal-seconds (Pa.s) against time and changes in frequency give an elasticity curve measured in Pascals (Pa) against time, as shown in Fig. 1. The clotting time (COT) can be obtained from the viscosity curve: COT1 represents the time to initiation of clot formation and COT2 the time when clot formation is complete and elasticity starts developing. COT2 is equivalent to ROTEM’s CT. The difference between COT2 and COT1 is a measure of clot progression. From the elasticity curve the slope, maximum elasticity (G’max; the maximum strength/stiffness of the clot) and clot strength reduction (Clot SR; fibrinolysis) can be determined. These correspond to ROTEM’s alpha angle, MCF or MCE and ML, respectively.

After addition of 25 μL of 0.5 M CaCl_2_ (MediRox AB) to 1000 μL of sample, coagulation was initiated with thromboplastin (the HepScreen1 reagent, MediRox AB). The FOR tracings analyzed were: COT1, COT2, slope, G’max and Clot SR.

### Statistical analysis

The ‘R’ Statistical environment (version 3.1.3: www.r-project.org) was used for statistical calculation and to create diagrams. Correlations were tested using Spearman’s test. The significances of differences between results for different levels of heparinization using the same LMWH and different LMWH’s at the same level of heparinization were tested using the Wilcoxon signed rank test. A P-value of <0.05 was considered significant. Friedman’s analysis of variance was used to detect significant differences in the distributions of results for enoxaparin and tinzaparin, taking into account inter-individual variation and differing concentrations of LMWH. Box and whisker diagrams were constructed using R’s boxplot function. Boxes span the interquartile range and the whiskers encompass the data point furthest from the box yet within 1.5 times the length of the box from the box.

## Results

Raw data of our results are available as a text file in ‘Additional file 1’.

### Measures of clot initiation were prolonged by increasing doses of LMWH

**Fig. 2:**
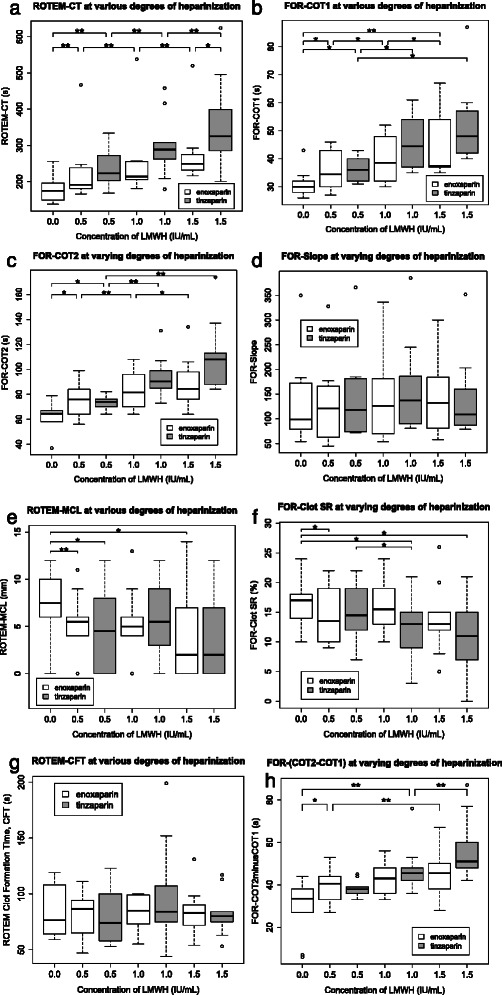
Box and whisker plots showing rotational thromboelastometry (ROTEM) and free-oscillation rheometry (FOR) results for enoxaparin and tinzaparin at varying concentrations. A brief explanation of the parameters follows: measures of clot initiation: ROTEM-CT (clot time) (**a**) and FOR-COT1 (**b**) and -COT2 (**c**). Measures of clot propagation: ROTEM-CFT (**g**); and FOR-(COT2-COT1) (**h**) and –Slope (**d**). Measures of fibrinolysis:: ROTEM-MCL (**e**) and FOR-ClotSR (**f**). *indicates a significant difference with p < 0.05. **indicates a significant difference with p < 0.01. Panels inside the figures both reflect inter- and intra-group comparisons

**Fig. 3:**
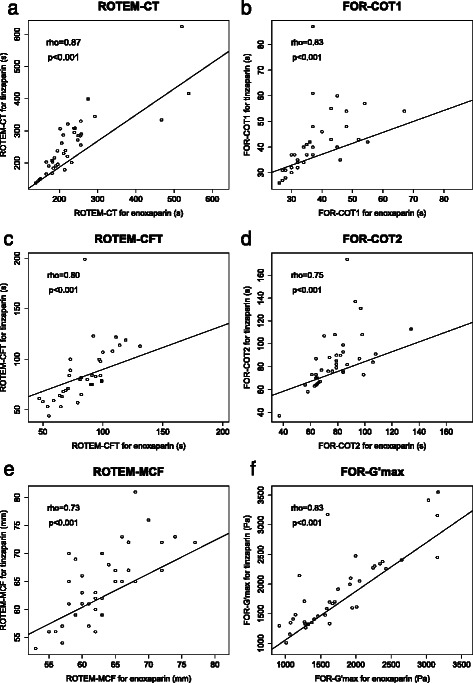
Scatter plot comparing ROTEM and FOR results at corresponding doses of enoxaparin and tinzaparin in IU/mL. Results are tightly and significantly correlated but tinzaparin has a stronger anticoagulative effect than enoxaparin at any given concentration. This is due to tinzaparin having a lower anti-FXa/anti-FIIa ratio than enoxaparin: for each unit of anti-FXa activity, tinzaparin has more anti-FIIa effect than enoxaparin. Rho: Spearman’s Rho: see methods section. A brief explanation of the parameters follows: measures of clot initiation: ROTEM-CT (clot time) (**a**) and FOR-COT1 (**b**) and -COT2 (**d**). Measures of clot propagation: ROTEM-CFT (**c**). Measures of clot structure: ROTEM-MCF (**e**) and FOR-G’max (**f**)

Measures of initiation of coagulation as assessed by ROTEM and FOR were significantly prolonged by increasing concentrations of both LMWH’s (ROTEM-CT, FOR-COT1 and FOR-COT2: see Table 1 and Fig. 2a-c), with significant correlation coefficients (Spearman’s Rho) of between 0.54 and 0.77, but there was a wide spread of results, with the lowest measured ROTEM-CT in the presence of 1.5 IU/mL tinzaparin being shorter than the longest ROTEM-CT in the control group (0 IU/mL tinzaparin). The two LMWH’s values of ROTEM-CT correlated to each other significantly, as did their values of FOR-COT1 and FOR-COT2 (see Figs. 3a,b and d).

**Table 1:** Rotational thromboelastometry (ROTEM) and free-oscillation rheometry (FOR) results at varying concentrations of enoxaparin and tinzaparin

	Manufacturer’s reference range	0 IU/mL	Enoxaparin	Enoxaparin	Enoxaparin	Tinzaparin	Tinzaparin	Tinzaparin	Enoxaparin vs Tinzaparin	Enoxaparin	Tinzaparin
			0.5 IU/mL	1.0 IU/mL	1.5 IU/mL	0.5 IU/mL	1.0 IU/mL	1.5 IU/mL	ANOVA❖	Spearman (Rho, P)	Spearman (Rho, P)
*ROTEM*											
CT (s)	100-240	178 ± 38	191 ± 89	214 ± 109	249 ± 94	223 ± 53	289 ± 84	326 ± 125	*P* < 0.01	0.62, *P* < 0.01	0.70, *P* < 0.01
CFT (s)	30-110	77 ± 23	87 ± 21	85 ± 16	83 ± 22	74 ± 27	84 ± 45	80 ± 21	P < 0.05	N/S	N/S
Angle (°)	70-83	75 ± 4	74 ± 4	75 ± 3	73 ± 4	75 ± 5	73 ± 5	73 ± 4	N/S	N/S	N/S
MCF (mm)	50-72	62 ± 5	61 ± 7	63 ± 5	63 ± 6	68 ± 7	64 ± 8	65 ± 6	N/S	N/S	N/S
ML (%)	<15	8 ± 4	6 ± 3	5 ± 4	2 ± 5	5 ± 4	6 ± 4	2 ± 4	N/S	−0.36, P < 0.05	N/S
*FOR (ReoRox)*											
COT1 (s)	20-35	30 ± 5	35 ± 7	39 ± 8	38 ± 11	36 ± 4	45 ± 9	48 ± 15	P < 0.01	0.58, P < 0.01	0.77, P < 0.01
COT2 (s)	30-90	65 ± 11	76 ± 13	82 ± 14	85 ± 20	74 ± 5	91 ± 16	108 ± 29	N/S	0.54, P < 0.01	0.84, P < 0.01
COT2-COT1 (s)	10-55	34 ± 13	41 ± 8	43 ± 9	46 ± 10	38 ± 4	46 ± 11	51 ± 15	P < 0.05	0.47, P < 0.01	0.79, P < 0.01
Slope (Pa/min)	45-145	99 ± 87	121 ± 82	126 ± 88	132 ± 76	118 ± 90	138 ± 94^*^	109 ± 88	P < 0.01	N/S	N/S
G’max (Pa)	770-2180	1629 ± 617	1777 ± 662	1831 ± 701	1612 ± 612	1656 ± 692	2069 ± 665	1615 ± 641	N/S	N/S	N/S
Clot SR (%)	10-25	17 ± 4	14 ± 5	16 ± 4	13 ± 6	15 ± 5	13 ± 6	11 ± 7	N/S	N/S	−0.41, P < 0.05

Results are presented as median ± SD. The significances of differences between individual concentrations, and between enoxaparin and tinzaparin at equal concentrations, are shown in Figs. 2 and 3, which display the results diagrammatically. ❖The significance of differences between results for enoxaparin and tinzaparin, corrected for concentration and individual, were assessed by Friedman’s analysis of variance (ANOVA). A brief explanation of the above tests follows. Measures of clot initiation: ROTEM-CT (clot time) and FOR-COT1 and -COT2. Measures of clot propagation: ROTEM-CFT and –alpha angle; and FOR-(COT2-COT1) and –Slope. Measures of clot structure: ROTEM-MCF and FOR-G’max. Measures of fibrinolysis: ROTEM-ML and FOR-Clot SR

*indicates a significant inter-class difference with *p*  < 0.05

### FOR-(COT2-COT1) was the only measure of clot propagation that showed a dose–response to LMWH

ROTEM-alpha angle, ROTEM-CFT and FOR-Slope were not affected by increasing concentrations of LMWH (see Table 1 and Fig. 2d and g). FOR-(COT2-COT1), which is the time delay between when viscosity starts to increase (COT1) and when elasticity starts to increase (COT2), was significantly prolonged by increasing doses of LMWH. The median (COT2-COT1) in the presence of 1.5 IU/mL tinzaparin and enoxaparin were 50 % and 30 % respectively longer than in the absence of added LMWH, giving correlation coefficients (Spearman’s Rho) of 0.47 and 0.79 respectively (*P* < 0.05). Although the differences between (COT2-COT1) for the two LMWH’s at each concentration were not significantly different, there was a significant whole-data difference in the results for tinzaparin and enoxaparin (see Table 1 and Fig. 2h). ROTEM-MCF and FOR-G’max for enoxaparin and tinzaparin showed good correlation (see Fig. 3e-f) but were not affected by increasing doses of either LMWH (see Table 1).

### ROTEM and FOR’s tests for clot lysis showed a tentative dose response

Neither our ROTEM-ML nor FOR-ClotSR results were outside the reference ranges for a normal level of fibrinolysis, and there were no significant differences between enoxaparin and tinzaparin at any concentration, or in ANOVA whole-data analysis. There was, however, a significant but weak negative correlation between the dose of enoxaparin and ROTEM-ML (**σ = −0.36,***P* < 0.05); and the dose of tinzaparin and FOR-Clot SR (**σ = −0.41,***P* < 0.05), but not between the dose of enoxaparin and FOR-Clot SR or tinzaparin and ROTEM-ML (see Table 1 and Fig. 2e and f).

## Discussion

Miyazaki et al. estimated that around 70 % of spinal hematomas occurring at the time of withdrawing an epidural catheter were related to abnormal coagulation, which challenges the dogma that monitoring of prophylactic LMWH is unnecessary in this setting [7]. Due to the difficulty and expense involved in conducting prospective studies on rare complications, it is very unlikely that such a study will ever be able to show that viscoelastic tests are reliable predictors of spinal haematoma.

The most common and well-documented clinical viscoelastic tests are thrombelastography (TEG®) and rotational thromboelastometry (ROTEM®). Less well-documented are free oscillation rheometry (FOR, ReoRox®) and Sonoclot® [12, 13, 21]. Although these assays measure the same aspects of coagulation and can detect both hypocoagulation and hypercoagulation, they differ in their mechanisms [21–23].

LMWH’s are a diverse group of antithrombotic molecules derived from unfractionated heparins (UFH) and have different structures and molecular weights (MW’s), which results in varying pharmacological features [24]. In this study coagulation following treatment with LMWH’s (enoxaparin and tinzaparin) was assessed using viscoelastic methods (thromboelastometry and FOR) to assess their potential to monitor treatment with LMWH’s.

Previous studies have shown varying abilities of viscoelastic devices to monitor treatment with LMWH’s [15, 16, 25–27] whereas UFH has been successfully monitored in healthy volunteers [27]. Louis et al. recently failed to show that the rate of deep vein thrombosis rate in trauma patients was reduced by using TEG tracings to titrate enoxaparin doses despite this leading to an increase in anti-FXa activity [28].

### Both ROTEM and FOR show a linear relationship between measures of clot initiation and concentration of LMWH, albeit with great inter-individual variation

We found that both LMWH substances prolonged both instruments’ measures of clot initiation in a significant dose-dependent manner, suggesting possible usefulness for postoperative monitoring. FOR measures both the time to initiation of increasing viscosity (COT1), reflecting early clot initiation and the time to increasing elasticity (COT2), which corresponds to ROTEM-CT. All these parameters increased significantly with increasing doses of LMWH’s, which is in agreement with previous research [16].

Whether the great inter-individual variation that we observed precludes these techniques use in monitoring LMWH’s depends on whether the results actually reflect the coagulation status of the patients or not: although methodological variation may account for some of the variation, the complex coagulation status occurring after major surgery is also likely to cause variation in patients’ response to any given dose of LMWH and it is possible that viscoelastic tests have a place in identifying ‘sensitive’ patients for whom a ‘normal dose’ is actually an overdose: preoperative malnourishment results in a reduced capacity to produce vitamin K dependent coagulation factors, and a major inflammatory response to surgery can be expected to cause shifts in plasma levels of coagulation factors. Shifts in fluid balance in the aftermath of haemorrhage with or without excessive transfusion can cause unpredictable variations in renal function and thereby pharmakokinetics. The postoperative state generally predisposes to hypercoagulation [29]. It is tempting to attribute the great inter-individual variation detected in this study exclusively to varying ‘postoperative factors’, but the results are actually in agreement with results from healthy volunteers given a direct factor Xa inhibitor by Casutt et al. in 2012 [30]. This is clearly an under-researched area of perioperative medicine and deserves more attention.

### Neither ROTEM nor FOR could detect that LMWH affected clot stability

We observed no significant correlation between the concentration of LMWH and maximum clot strength (ROTEM-MCF and CFT, and FOR-Slope and G’max), see Fig. 2 and Table 1. This confirms previous work by Feuring et al., who observed that ROTEM-MCF was only affected by supratherapeutic levels of dalteparin; but is in contrast to Gerotziafas et al. who found that therapeutic doses of enoxaparin did indeed affect TEG-MA (thrombelastography maximum amplitude, corresponds to ROTEM-MCF) in healthy volunteers [25, 31]. LMWH consistently impedes clot initiation as measured by viscoelastic tests but not clot propagation or structure, but this does not necessarily mean that LMWH does not affect clot propagation *in vivo* since both the *ex vivo* viscoelastic tests discussed in this article are flawed by the fact that they monitor coagulation in a stagnant container. *In vivo* coagulation takes place within or beside blood vessels in which there is blood flow. If clot initiation is too slow in a microenvironment where there is constant flow, the clot may be ‘washed away’ before it has even formed. In contrast, even a very slow-forming clot in a viscoelastic test container is able to contribute to the cell mediated positive-feedback loops that maintain propagation.

We had hypothesised that FOR might be more sensitive to LMWH’s possible attenuations of clot propagation and maximum amplitude, but this could not be confirmed by our results. Our hypothesis was based on the knowledge that the shear forces applied by rotational thromboelastometry are known to exceed the linear viscoelastic properties of clots and may therefore in themselves weaken the developing clot [32]. FOR, however, does not apply shear force to the sample: it applies a short oscillation every 2.5 s instead, which allows measurement of both viscosity and elasticity, and should also disturb the clot less than ROTEM [33]. Other differences between the techniques that may lead to differing patterns of contact activation are that the reagents used to initiate coagulation are different: ROTEM® uses thromboplastin phospholipid and ellagic acid whereas ReoRox® uses thromboplastin alone, potentially resulting in different patterns of activation. The surfaces in the ROTEM® chamber are plastic while ReoRox® is gold-plated, which may affect initiation of coagulation and reduce the tendency of the clot to loosen from the cup wall giving a false impression of fibrinolysis.

### The dose-effect observed on fibrinolysis was only tentative, and surprisingly suggested that increasing doses of LMWH’s decreased fibrinolysis

Although the statistical significance of the dose-effect of LMWH’s on measures of fibrinolysis were only tentative, Fig. 2e and f show a negative dose–response that may deserve further investigation. Previous findings suggest that LMWH’s increase rather than decrease fibrinolysis [34]: it is an interesting hypothesis that the postoperative coagulative environment may provide conditions where the inverse is true.

### Tinzaparin is more potent than enoxaparin and the two LMWH’s measureable effects in this study are linearly correlated. We again question anti-FXa activity’s ‘gold standard status’ for monitoring LMWH

We found significant correlations between tinzaparin and enoxaparin for several ROTEM and FOR parameters. However, all the parameters for which a dose–response could be demonstrated in this study showed that tinzaparin had a stronger anticoagulant effect than a corresponding dose of enoxaparin in international units per millilitre (see Fig. 2 and 3). This is in line with our previous findings where tinzaparin has been shown to prolong aPTT and impede thrombin generation to a greater degree than enoxaparin, and as explained previously is due to tinzaparin having a lower anti-FXa/anti-FIIa ratio than enoxaparin. If the two LMWH’s are dosed in equal units of anti-FXa activity (‘international units’), the tinzaparin will have a stronger overall anticoagulant effect due to the anti-IIa activity which accompanies each unit of anti-FXa activity [11]. There is also evidence that UFH and LMWH’s with larger molecular weight (>2 kDa) exert an anticoagulant effect through plasma tissue factor pathway inhibitor [35].

At many institutions, including our own institution, the anti-FXa activity assay has become the clinical ‘gold standard’ for monitoring LMWH’s. Although anti-FXa activity is likely a reliable measure of LMWH concentration [36], we would advise against relying on this assay alone to titrate the dose of LMWH: we suggest that several assays (anti-FXa, aPTT, antithrombin, viscoelastic tests, possibly thrombin generation) should be run concurrently and in series. Laboratory results should be combined with clinical judgement to dose LMWH’s in patients at risk of thromboembolic or haemorrhagic complications, particularly in patients where haemorrhage could be catastrophic, such as those whose epidural catheter is due to be withdrawn. This is not particularly new: in 2009 Van *et al.* observed that thrombelastography was a better predictor of deep vein thrombosis than anti-FXa activity in trauma and surgical patients [14].

### Limitations of this study

There are some limitations to this study: it is a small *in vitro* dose–response study and should thus be viewed as a pilot study with low specificity. Since all our patients are given LMWH to prevent postoperative thromboembolism, it was not possible to run tests on a control group that had been exposed to major surgery but not LMWH. While preoperative ‘baseline’ analyses could have been taken, they could potentially have been misleading since LMWH is only one of the factors affecting postoperative coagulation.

A criticism of the method could be that we did not test for the samples’ haematocrits and adjust the doses of LMWH accordingly: a lower haematocrit means a greater fraction of plasma in the sample, and therefore a greater ‘volume of distribution’ for the LMWH that we added. We nevertheless decided to administer LMWH to our samples in standard doses because this is what happens in clinical practice: LMWH is either prescribed in standard doses or by weight, which are rarely adjusted for renal function or haematocrit.

## Conclusions

Both ROTEM and FOR showed clot-initiation to be prolonged by increasing doses of both LMWH’s albeit with significant inter-individual variation, which may preclude their use in monitoring LMWH in the postoperative period: it is unclear from this study whether the inter-individual variation was due to methodological variation or ‘true’ pharmacodynamic variation. The dose–response was, as expected, significantly greater for tinzaparin than enoxaparin at equivalent doses of anti-Xa activity. We could not confirm our hypothesis that FOR could measure LMWH’s effects on other measures of coagulation more sensitively than rotational thromboelastometry. More research is needed before any conclusion can be made about the superiority of ROTEM or FOR in individualizing thromboprophylactic or therapeutic therapy with LMWH.

## Additional file

Additional file 1: **Raw data of our results.** (TXT 3 kb)

## Abbreviations

ANOVA: Analysis of variance
anti-FXa: Anti-factor Xa
aPTT: Activated partial thromboplastin time
CFT: Rotational thromboelastometry® clot formation time
ClotSR: ReoRox® clot strength Reduction
CT: Rotational thromboelastometry® clotting time
DVT: Deep vein thrombosis
FOR: Free-oscillation rheometry
G’max: ReoRox® maximum elasticity
IU: International Unit
LMWH: Low molecular weight heparin
MA: TEG® maximum amplitude
MCE: Rotational thromboelastometry® maximum clot elasticity (calculated as 100 × MCF÷(100-MCF).
MCF: Rotational thromboelastometry® maximum clot firmness
ML: ROTEM® maximum clot lysis
MW: Molecular weight
PE: Pulmonary embolism
PLT: Platelet count
PT-INR: Prothrombrin time international normalized ratio
ROTEM®: Rotational thromboelastometry
TEG®: Thrombelastography
UFH: Unfractionated heparin
VHT: Viscoelastic haemostatic test
